# Evaluating the Effect of Maternal Opioid Maintenance Dose on the NOWS-COS Outcome Criteria: A Pilot Study

**DOI:** 10.1177/00099228241246647

**Published:** 2024-04-17

**Authors:** Danielle Smith, Rhea Sullivan, Emma C. Allen, Sandeep Pradhan, Junjia Zhu, Christiana N. Oji-Mmuo

**Affiliations:** 1 Division of Neonatal-Perinatal Medicine, Department of Pediatrics, Penn State College of Medicine, Hershey, PA, USA; 2 Department of Public Health Sciences, Penn State College of Medicine, Hershey, PA, USA; 3 Department of Pediatrics, Dell Medical School, The University of Texas at Austin, Austin, TX, USA

**Keywords:** NOWS, MME, buprenorphine, developmental, delay, opioid

## Abstract

This retrospective cohort study included 77 mother-infant dyads that delivered term pregnancies at a single tertiary care institution. The primary objective was to investigate whether maternal dose of opioid maintenance therapy during pregnancy affects infant outcomes. All infants had prenatal exposure to opioid maintenance therapies. Maternal dose was converted into morphine milligram equivalents (MMEs) and stratified into high- (MME >1000 mg) and low-dose groups (MME ≤1000 mg). Associations between infant outcomes and MME dosage were examined using Wilcoxon rank-sum and Fisher’s Exact tests. Days to symptom control were significantly higher in the high MME group (5 days vs 2.8 days, *P* = .016). Rates of developmental delay at 24 months were higher in the high MME group (21.2% vs 4.5%, *P* = .0335). Maternal MME did not predict need for NOWS treatment. Higher MME-exposed infants should have optimized nonpharmacologic interventions for consolation and be increasingly observed for signs of developmental delay.

## Introduction

Neonatal opioid withdrawal syndrome (NOWS) is a condition characterized by autonomic and central nervous system hyperactivity that occurs when a newborn experiences withdrawal from prenatal opioid exposure.^1^ Maternal opioid use disorder (OUD) is the leading cause of NOWS. As a result of the ongoing opioid epidemic, there has been an increase in OUD in pregnancy, and subsequently, a rise in NOWS incidence.^2^ The growth of OUD, however, is significantly higher than the incidence of NOWS, which confirms the observation that not all opioid-exposed newborns develop NOWS.^3^ Despite a growing body of research studying NOWS outcomes, there is a lack of consensus regarding how maternal factors may influence such outcomes.^4^

The standard of care for OUD is to start medication assisted therapy with either methadone or buprenorphine.^5^ Medication for OUD in pregnancy has been shown to reduce risks of illness or death of the mother and infant during gestation.^6^ Whereas many studies have focused on associations between types of medications for OUD and NOWS infant outcomes,^7-9^ fewer have investigated the morphine milligram equivalent (MME) dose relationship on opioid-exposed newborns.^10^ Converting maternal medication for OUD dosages to MME when studying NOWS outcomes is critical to standardizing dose effects across therapies.^10^

The primary objective of this study was to evaluate the association of maternal MME dose (stratified by high vs low) with infant outcomes in those exposed to methadone, buprenorphine, or buprenorphine-naloxone during at least the last month of pregnancy. To promote homogeneity and generalizability between other NOWS outcome studies, we chose to follow the standard 13 outcomes proposed in the 2020 Neonatal Opioid Withdrawal Syndrome Core Outcome Set (NOWS-COS) criteria by Kelly et al.^11^ To our knowledge, this is the first published study following the NOWS-COS criteria and the first study to associate these outcomes with maternal MME dose. We hypothesize that infants exposed to higher maternal MME doses will have poorer short- and long-term clinical outcomes than those exposed to less maternal MME doses.

## Methods

### Design and Participants

This retrospective cohort study was granted an exemption waiver for informed consent by the Institutional Review Board. This research was performed in accordance with the Declaration of Helsinki. Study data were collected and managed using REDCap electronic data capture tools hosted at our institution.^12^ REDCap (Research Electronic Data Capture) is a secure, web-based application designed to support data capture for research studies, providing (1) an intuitive interface for validated data entry; (2) audit trails for tracking data manipulation and export procedures; and (3) automated export procedures for seamless data downloads to common statistical packages procedures for importing data from external sources.

The electronic medical record was queried to extract mother-infant dyad data with infants born between January 2010 and December 2020 at ≥37 weeks gestation and prenatal exposure to methadone, buprenorphine, or buprenorphine-naloxone. Exclusion criteria included infants born preterm (<37 weeks gestation), prenatal exposure to non-opioid, non-prescription drugs, respiratory distress requiring >2 L/min nasal cannula, maternal polypharmacy other than nicotine, cardiorespiratory instability, and infants with sepsis or other infection. Infants discharged home with opioid medications were also excluded due to the difficulty of monitoring response, such as time to symptom control. Initially, we identified 224 mother-infant dyads. Of these, 147 were excluded from the study due to prenatal exposure to non-opioid or non-prescription drugs, prematurity, cardiorespiratory instability, and discharge home with medication. The remaining population included 77 mother-infant dyads.

Maternal doses of opioids (in mg/day) were converted into MME and then dichotomized into high-dose (MME > 1000mg) and low-dose groups (MME ≤1000 mg). This cut off was determined based on similar cut offs used in the literature^13^ and natural groupings formed in our collected data. Other maternal factors explored included marital status, delivery type, maternal length of opioid use during pregnancy, duration of lifetime opioid use, and nicotine use. The dependent variables explored in this study were based on the NOWS-COS criteria and included pharmacologic treatment, the total dose of opioid treatment, duration of treatment, adjuvant therapy, feeding difﬁculties, consolability, time to adequate symptom control, parent-infant bonding, duration of time the neonate spent in the hospital, breastfeeding, weight gain at hospital discharge, readmission to hospital for withdrawal, and the presence of developmental delays by 24 months of age. Consolability was defined as the ability to control symptoms with either non-pharmacologic interventions and/or ability to wean morphine within 48 hours. Parent-infant bonding was assessed by observing parental involvement in care as recorded in the electronic medical record. Developmental delay was assessed at 24 months of age by the infants’ pediatrician as documented in the electronic medical record.

### Statistical Analysis

The patient demographics and basic clinical measurements at birth were summarized using descriptive statistics, such as means and standard deviations for continuous variables and counts and percentages for categorical variables. The 13 primary outcomes measured from the 2020 NOWS-COS study are a mixture of continuous quantitative variables and categorical variables. The bivariate associations between these 13 outcomes and maternal MME dose were examined using nonparametric Wilcoxon rank-sum test for continuous outcome variables and Fisher’s Exact test for categorical outcome variables. All analyses were performed using the statistical software SAS version 9.4 (SAS Institute Inc., Cary, NC, USA). All tests were 2-sided using statistical significance level 0.05.

It was initially estimated that about 200 patient records could be extracted over a 10-year period at our institute, yielding a statistical power of >94% to detect a standardized effect size of 0.5. With a reduced sample size of 77 dyads, we were able to detect a larger standardized effect size of 0.67 with about 82% statistical power. Due to the relatively small sample size and novelty, this study is exploratory in nature. Thus, no adjustment on statistical significance level was made for multiple testing.

## Results

Patient demographic information of the 77 mother-infant dyads is shown in Table 1. Outcomes are stratified in Table 2 by maternal MME groups. A total of 44 mother-infant dyads were stratified to the low-dose category (≤1000 mg) and 33 mother-infant dyads were stratified to the high-dose category (>1000 mg).

**Table 1.** Patient Demographics.

Post menstrual age (weeks)	
N	77
Mean (SD)	39.1 (1.22)
Postnatal age at discharge (days)	
N	76
Mean (SD)	13.5 (12.25)
Gender, n (%)	
Male	39 (50.6%)
Female	38 (49.4%)
Birth weight (g)	
N	77
Mean (SD)	3191.7 (447.52)
Infant race, n (%)	
White	68 (93.2%)
Hispanic	3 (4.1%)
Bi-racial	2 (2.7%)
Maternal race, n (%)	
White	69 (93.2%)
Hispanic	2 (2.7%)
Bi-racial	3 (4.1%)
Marital status, n (%)	
Married	10 (13.5%)
Not married, living with partner	24 (32.4%)
Single	31 (41.9%)
Divorced/separated	8 (10.8%)
Other	1 (1.4%)
Apgar 1 min, n (%)	
<7	5 (6.6%)
≥7	71 (93.4%)
Apgar 5 mins, n (%)	
≥7	72 (100.0%)
Birth Length (cm)	
N	76
Mean (SD)	49.8 (2.30)
Discharge length (cm)	
N	66
Mean (SD)	50.1 (3.16)
Birth head circumference (cm)	
N	75
Mean (SD)	33.7 (1.49)
Discharge head circumference (cm)	
N	67
Mean (SD)	34.6 (1.70)
Median	34.3
Range	31.0 - 39.5
Discharge weight (g)	
N	77
Mean (SD)	3190.4 (666.53)
Discharge weight z-score	
N	75
Mean (SD)	−1.1 (0.94)
Prenatal care, n (%)	
No	12 (16.0%)
Yes	63 (84.0%)
Maternal length of opioid use during pregnancy (months)	
N	77
Mean (SD)	8.9 (0.57)
Maternal duration of lifetime use of opioids, n (%)	
Greater than 1 year	77 (100.0%)
Maternal type of opioid, n (%)	
Methadone	49 (63.6%)
Buprenorphine	21 (27.3%)
Buprenorphine-naloxone	7 (9.1%)
Maternal nicotine use during pregnancy, n (%)	
No	20 (26.0%)
Yes	57 (74.0%)

**Table 2.** Infant Outcomes Stratified by Maternal MME Categories.

	Low maternal MME, ≤1000 mg (N = 44)	High maternal MME, >1000 mg (N = 33)	*P* value
First-line and second-line therapy, n (%)			.3192^a^
1st line + adjuvant therapy	1 (5.9%)	3 (20.0%)	
Morphine (first line)	16 (94.1%)	12 (80.0%)	
Total morphine dose (mg/kg)			.2127^b^
N	17	15	
Mean (SD)	4.9 (4.21)	7.2 (6.57)	
Length of therapy (days)			.2506^b^
N	36	25	
Mean (SD)	8.3 (11.41)	12.3 (14.85)	
Consolability, n (%)			.0531^a^
No	6 (13.6%)	11 (33.3%)	
Yes	38 (86.4%)	22 (66.7%)	
Difficulty feeding, n (%)			.2773^a^
No	41 (93.2%)	28 (84.8%)	
Yes	3 (6.8%)	5 (15.2%)	
Need for pharmacologic therapy, n (%)			.6422^a^
No	27 (61.4%)	18 (54.5%)	
Yes	17 (38.6%)	15 (45.5%)	
Time to symptom control (days)			.0164^b^
N	17	15	
Mean (SD)	2.8 (2.07)	5.0 (3.74)	
Parent-infant bonding, n (%)			.5401^a^
No	6 (13.6%)	7 (21.2%)	
Yes	38 (86.4%)	26 (78.8%)	
Length of hospitalization (days)			.6435^b^
N	44	33	
Mean (SD)	12.1 (10.60)	14.4 (13.36)	
Breast Feeding, n (%)			.2005^a^
No	14 (31.8%)	6 (18.2%)	
Yes	30 (68.2%)	27 (81.8%)	
Weight gain from birth to discharge (g)			.4131^b^
N	44	33	
Mean (SD)	−35.4 (381.85)	−24.4 (697.46)	
Readmission to hospital, n (%)			.4286^a^
No	44 (100.0%)	32 (97.0%)	
Yes	0 (0.0%)	1 (3.0%)	
Development delays, n (%)			.0335^a^
Delay	2 (4.5%)	7 (21.2%)	
No delay	42 (95.5%)	26 (78.8%)	

a Fisher Exact *P* value; ^b^Wilcoxon rank sum *P* value.

Maternal MME grouping was not associated with need for pharmacologic therapy, need for second-line pharmacologic intervention, total morphine dose, length of therapy, difficulty feeding, parent-infant bonding, length of hospitalization, breastfeeding, weight gain, or readmission for NOWS (Table 2). Infants in the low-dose group were more easily consoled compared to those in the high-dose group, though this was not statistically significant (*P* = .053). The mean number of days to symptom control between the 2 groups, however, were significantly different. In the low-dose group, the mean number of days to withdrawal symptom control was 2.8 days, whereas in the high-dose group, it was 5 days (*P* = .0164). In addition, infants in the high-dose group were more likely to have developmental delays identified at 24 months of age (21.2% vs 4.5%, *P* = .0355).

These outcomes were also investigated over 2 time periods (2010-2015 and 2016-2020) to account for variances in practice over time and are demonstrated in Tables 3 and 4. The outcomes over these 2 time periods were similar to the 10-year period outcomes as shown in Table 2. One notable difference was the improvement in parent-infant bonding in the later time group (*P* = .0118).

**Table 3.** Patient Outcomes Stratified by Maternal MME Categories for Group 1 (2010-2015).

	Low maternal MME, ≤1000 mg (N = 27)	High maternal MME, >1000 mg (N = 19)	*P* value
First-line and second-line therapy, n (%)			.1637^a^
First line + adjuvant therapy	0 (0.0%)	2 (25.0%)	
Morphine (1st line)	11 (100.0%)	6 (75.0%)	
Total morphine dose (mg/kg)			.5915^b^
N	11	8	
Mean (SD)	4.8 (3.18)	6.9 (6.40)	
Length of therapy (days)			.9546^b^
N	19	14	
Mean (SD)	9.9 (10.38)	11.6 (15.68)	
Consolability, n (%)			.2772^a^
No	4 (14.8%)	6 (31.6%)	
Yes	23 (85.2%)	13 (68.4%)	
Difficulty feeding, n (%)			1.0000^a^
No	26 (96.3%)	18 (94.7%)	
Yes	1 (3.7%)	1 (5.3%)	
Need for pharmacologic treatment, n (%)			1.0000^a^
No	16 (59.3%)	11 (57.9%)	
Yes	11 (40.7%)	8 (42.1%)	
Time to symptom control (days)			.0343^b^
N	11	8	
Mean (SD)	2.6 (1.57)	4.6 (2.88)	
Parent-infant bonding, n (%)			.4395^a^
No	6 (22.2%)	2 (10.5%)	
Yes	21 (77.8%)	17 (89.5%)	
Length of hospitalization (days)			.9275^b^
N	27	19	
Mean (SD)	12.5 (9.92)	13.3 (13.67)	
Breast feeding, n (%)			.0442^a^
No	11 (40.7%)	2 (10.5%)	
Yes	16 (59.3%)	17 (89.5%)	
Weight gain from birth to discharge (g)			.7632^b^
N	27	19	
Mean (SD)	−21.1 (337.11)	42.4 (573.26)	
Readmission to hospital, n (%)			
No	27 (100.0%)	19 (100.0%)	
Development delays, n (%)			.2133^a^
Delay	2 (7.4%)	4 (21.1%)	
No Delay	25 (92.6%)	15 (78.9%)	

a Fisher Exact test *P* value; ^b^Wilcoxon rank sum test *P* value.

**Table 4.** Patient Outcomes Stratified by Maternal MME Categories for Group 2 (2016-2020).

	Low maternal MME, ≤1000 mg (N = 17)	High maternal MME, >1000 mg (N = 14)	*P* value
First-line and second-line therapy, n (%)			1.0000^a^
First line + adjuvant therapy	1 (16.7%)	1 (14.3%)	
Morphine (first line)	5 (83.3%)	6 (85.7%)	
Total morphine dose (mg/kg)			.2246^b^
N	6	7	
Mean (SD)	5.1 (6.04)	7.7 (7.24)	
Length of therapy (days)			.1015^b^
N	17	11	
Mean (SD)	6.5 (12.52)	13.2 (14.43)	
Consolability, n (%)			.1975^a^
No	2 (11.8%)	5 (35.7%)	
Yes	15 (88.2%)	9 (64.3%)	
Difficulty feeding, n (%)			.3697^a^
No	15 (88.2%)	10 (71.4%)	
Yes	2 (11.8%)	4 (28.6%)	
Need for pharmacologic treatment, n (%)			.4809^a^
No	11 (64.7%)	7 (50.0%)	
Yes	6 (35.3%)	7 (50.0%)	
Time to symptom control (days)			.2445^b^
N	6	7	
Mean (SD)	3.2 (2.93)	5.4 (4.76)	
Parent-infant bonding, n (%)			.0118^a^
No	0 (0.0%)	5 (35.7%)	
Yes	17 (100.0%)	9 (64.3%)	
Length of hospitalization (days)			.3927^b^
N	17	14	
Mean (SD)	11.5 (11.88)	15.8 (13.29)	
Breast feeding, n (%)			.6705^a^
No	3 (17.6%)	4 (28.6%)	
Yes	14 (82.4%)	10 (71.4%)	
Weight gain from birth to discharge (g)			.1835^b^
N	17	14	
Mean (SD)	−57.9 (454.22)	−115.1 (852.67)	
Readmission to hospital, n (%)			.4516^a^
No	17 (100.0%)	13 (92.9%)	
Yes	0 (0.0%)	1 (7.1%)	
Development delays, n (%)			.0810^a^
Delay	0 (0.0%)	3 (21.4%)	
No Delay	17 (100.0%)	11 (78.6%)	

a Fisher Exact test p-value; ^b^Wilcoxon rank sum test p-value.

## Discussion

The few published studies that have associated maternal medications for OUD with NOWS outcomes have focused on stratifying by drug type, and then by dose.^14^ Converting maternal doses to MME introduces a standardized way of quantifying prenatal exposures.^10^ An outcome from this study was that maternal MME dose did not predict the need for NOWS pharmacologic treatment. This finding recapitulates a previous report from Patrick et al^10^ that neonatal abstinence syndrome risk is not associated with cumulative prenatal MME exposure, without adjusting for other factors. However, we present the first evidence that differences in maternal MME dose may hold clinical significance for infants treated for NOWS. We showed that infants exposed to a lower maternal MME dose achieved symptom control faster, at a mean of 2.8 days compared to infants with high maternal MME exposure at 5 days. In addition, infants exposed to low maternal MME were more easily consoled, though these results were not statistically significant. Similarly, Lappen et al^14^ found that more severe neonatal withdrawal symptoms were associated with higher levels of maternal methadone at the time of delivery, though this has been shown inconsistently across other studies.^13^

We conclude that nonpharmacologic interventions should be optimized for NOWS infants exposed to higher maternal MME. Since more time is spent identifying the maintenance phase dose required for symptom control, these infants will experience prolonged discomfort compared to their lower maternal MME exposure cohort. This finding is timely; a recent NOWS outcome study during the COVID-19 pandemic reported decreased rooming-in due to hospital isolation procedures.^15^ Measures of nonpharmacologic therapy modalities outlined in the NOWS-COS guidelines include parent-infant bonding, breastfeeding, consolability, and feeding difficulties.^11^ Given the highly documented evidence that these are critical for optimal NOWS infant consolability, we reiterate the importance of applying these by supporting maternal lactation efforts and parental involvement in the care of higher maternal MME-exposed infants.^16^

It is thought that infants treated for NOWS are at an increased risk of developmental delays through childhood,^2,17,18^ though there have been both inconsistent results and an inadequate quantity of studies.^8,19^ In this study, we present the first evidence suggesting maternal MME is a risk factor for presence of developmental delays by 24 months in infants with NOWS. We conclude that infants prenatally exposed to higher MME be increasingly observed for referral to early invention providers prior to 2 years of age.

This study was subject to several limitations. This was a retrospective cohort study, and the specific management of each infant with NOWS may have differed. Data were collected from mother-infant dyads that delivered between January 2010 to December 2020 at a single institution. Over the course of those 10 years, the frequency of modified Finnegan score assessments, interpretation of modified Finnegan scores, and overall management of infants with NOWS evolved. For example, before the hospital expansion in 2020, the NICU at this institution was not equipped with private rooms for all infants with NOWS and their families. Research has shown that decreasing stimulation and other environmental controls has positive effect on infants with NOWS,^16^ so it could be possible that over the course of the study timeline evolving methods, especially non-pharmacological methods, used in the hospital may have affected the outcomes in this study. In addition, this institution joined the state’s perinatal collaborative group to improve care of infants prenatally exposed to substances after 2016. This was reflected in the breakdown by time period with an increase in parent-infant bonding in the later time group. We were unable to obtain Bayley scores on the infants included at 24 months of age due to inconsistent follow-up in the NICU Developmental Clinic. The developmental delays reported are based on primary care visits for patients who remained in the health care system and did not encompass all infants in this study. Finally, we recognize that the sample size was small (n = 77) with only 33 infants required pharmacologic NOWS treatment. Further studies should be replicated with larger sample sizes across multiple institutions to assess generalizability.

In conclusion, we have presented the first evidence of maternal MME dose being associated with time to NOWS infant symptom control and risk of developmental delays by 24 months of age. We propose that non-pharmacologic interventions for consolability should be optimized for infants exposed to higher maternal MME doses. Maternal MME did not predict need for pharmacologic treatment of NOWS. In addition, we propose that infants with higher prenatal MME exposure be increasingly observed for developmental delays prior to 24 months of age to allow for prompt early intervention services.

## Author Contributions

DS: Contributed to design; acquisition, analysis, and interpretation of data; drafted manuscript; critically revised manuscript; and gave final approval. RS: Contributed to acquisition and interpretation of data; drafted manuscript; critically revised manuscript; and gave final approval. ECA: Contributed to acquisition and interpretation of data; drafted manuscript; critically revised manuscript; and gave final approval. SP: Contributed to analysis and interpretation of data; drafted manuscript; critically revised manuscript; and gave final approval. JZ: Contributed to design; analysis and interpretation of data; drafted manuscript; critically revised manuscript; and gave final approval. CNOM: Contributed to conception and design; acquisition, analysis, and interpretation of data; drafted manuscript; critically revised manuscript; and gave final approval. All authors agree to be accountable for all aspects of work ensuring integrity and accuracy.
